# Hodgkin Lymphoma in Childhood: Clinicopathological features and therapy outcome at 2 Centers From a Developing Country

**DOI:** 10.1097/MD.0000000000000670

**Published:** 2015-04-17

**Authors:** Laila M. Sherief, Usama R. Elsafy, Elhamy R. Abdelkhalek, Naglaa M. Kamal, Rabab Elbehedy, Tamer H. Hassan, Hanan S. Sherbiny, Mohamed R. Beshir, Safaa H. Saleh

**Affiliations:** From the Departments of Pediatrics, Faculty of Medicine, Zagazig (LMS, URE, ERA, RE, THH, HSS, MRB, SHS); Cairo Universities (NMK); and Benha Specialized Pediatric Hospital, Benha, Egypt (LMS, ERA).

## Abstract

Hodgkin lymphoma (HL) accounts for 5% to 6% of all childhood cancer. It displays characteristic epidemiological, clinical, and pathological features according to various geographic areas. We aimed to assess the epidemiological aspects, clinicopathological features, and treatment outcome of pediatric HL treated at 2 Egyptian centers: Zagazig University Pediatric Oncology Unit and Benha Special Hospital Pediatric Oncology Unit.

We carried a cross-sectional retrospective study by reviewing medical records for all patients admitted with the diagnosis of HL over 8 years in 2 oncology units during the period from January 2004 to January 2012.

Age of the patients at presentation ranged from 3 to 14 years (median 6 years) and male: female ratio 1.7:1. Lymphadenopathy was the most common presentation (96.6%). Mixed cellularity subtype was dominant (50.8%), followed by nodular sclerosis (28.9%), lymphocyte-rich (18.6%) with lymphocyte depletion being the least dominant (1.7%). More than half of patients (55.9 %) had advanced disease (Ann Arbor stage III/IV disease). The duration of follow-up ranged from 5 to 87 months (mean 39.8 ± 24.1 months). The 5-year overall survival and event-free survival for patients were 96.6% and 84.7% respectively.

In Egypt, HL occurs in young age group, with a higher incidence of mixed cellularity subtype and advanced disease. None of the clinical, epidemiological, or pathological characteristics had a significant association with the overall survival. The outcomes of HL in our 2 centers were satisfactory approaching the international percentage.

## INTRODUCTION

Hodgkin lymphoma (HL) accounts for 5% to 6% of all childhood cancer. It displays characteristic epidemiologic, clinical, and pathological features according to various geographic areas, particularly according to the socioeconomic level of a given country.^1–7^ In developing countries, HL appears more during childhood and its incidence decreases with age, whereas in developed countries, young children are rarely affected by HL in contrast with young adults in which incidence increase with age.^5^ The treatment of HL is one of success stories of modern medicine. There is unified pathologic classification schema, a noninvasive staging evaluation, and an increasing sophisticated approach to therapy with risk and response-adapted therapies in pediatric and adult populations.^8^ The 5-year event-free survival (EFS) in childhood and adolescence exceeds 90% for patients with early-stage^9^ and 70% to 80% for those with advanced-stage disease.^10–11^

Although publications on the disease from Western countries and rest of the world are numerous in medical literature, very few data are available in Africa.^12^

It was the scarcity of existing data in the literature illustrating the behavior of HL among children of our region that challenged us to carry out this study, with the intention of describing the clinical and epidemiologic characteristics of Egyptian HL patients, assessing their survival and searching for possible prognostic association.

## PATIENTS AND METHODS

We retrospectively reviewed the records of children and adolescents with HL treated at 2 Egyptian Pediatric Oncology Units, Zagazig University Hospital and Benha Specialized Pediatric Hospital, during the period from January 2004 to January 2012. The study was approved by the research and ethical committees of the contributing hospitals. Patients’ records were searched for demographic data, history details, examination findings specially nodal and extranodal involvement, investigations results, methods of establishing diagnosis, treatment courses, and follow-up. The enrolled patients were fulfilling the following inclusion criteria: age ≤18 years, HL diagnosis established by both characteristic histopathological findings and immunophenotypic studies and was subclassified according to the World Health Organization Classification. Anticancer treatment courses and follow-up were totally completed at the contributing centers. Diagnostic evaluation included biopsy of clinically involved lymph node or mass, computed tomography scans of neck, chest, abdomen, and pelvis, and bone marrow (BM) aspirations and biopsies and bone scintigraphy for selected cases.

The patients were subdivided into 3 risk groups according to presence or absence of risk factors.

Risk factors of HL (at least 2 factors are necessary):

1. Number of involved nodal sites >3.
2. Presence of extranodal disease.
3. Bulky mediastinal disease (≥0.35 of maximal intrathoracic diameter).
4. Initial erythrocyte sedimentation rate (ESR) >50 mm/h.
5. Unfavorable histology (lymphocyte depletion).
6. Presence of (B) symptoms.

Low-risk patients included all cases with stage I and II without risk factors.

Intermediate risk included cases with stages I and II with risk factors as well as stage IIIA1 irrespective of risk factors.

High risk included all cases with stages III A2, IIIB, and IV irrespective of risk factors.

### Treatment Protocol

Low-risk patients were treated with 4 cycles of doxorubicin, bleomycin, vinblastine, and dacarbazine (ABVD). Intermediate-risk patients were treated with 6 cycles of chemotherapy of ABVD. High-risk patients were treated with 8 cycles of chemotherapy of ABVD with no radiotherapy.

All stages I, II, and IIIA1 nonbulky patients who achieve a complete remission following chemotherapy will receive involved field radiotherapy (2100cGY).

Stage I–IIIA patients considered to have partial response after chemotherapy should receive 2100 cGy involved field radiation (IFR) with boost dose of 1400 cGY to residual disease sites (high-dose IFR; total 3500cGY).

Complete remission (CR) was achieved in all alive patients (2 patients died) and both chemotherapy and radiotherapy were well tolerated and without significant toxicity. Patients experienced occasional myelosuppression, mucositis, and nausea and vomiting managed by supportive care. Seven patients relapsed. All received high-dose chemotherapy ICE (ifosamide, caboplatin, etoposide) followed by autologous stem cell transplantation. All patients were alive in second complete remission. Two patients died of refractory metastatic disease.

## STATISTICAL ANALYSIS

Data were statistically described in terms of mean ± standard deviation (±SD), and range, or frequencies (number of cases) and percentages when appropriate. Survival analysis was done for the different outcome measures using Kaplan–Meier statistics calculating the mean and median survival time for each group with their 95% confidence interval (CI) and the corresponding survival graphs. *P* values <0.05 were considered statistically significant. All statistical calculations were done using computer program SPSS (Statistical Package for the Social Science; SPSS Inc, Chicago, IL) version 15 for Microsoft Windows.

## RESULTS

During the period between January 2004 and January 2012, 59 patients with HL were admitted to the Pediatric Oncology Units of Zagazig University Hospital and Benha Specialized Pediatric Hospital. Table 1 demonstrates demographic data of the included patients wherein the age at diagnosis ranged from 3 to 14 years with median 6 years. Most cases (54.2%) were diagnosed in children aged 5 to 10 years followed by children aged 10 to 15 years (23.7%), and 22% occurred in children younger than 5 years. The patient's population of HL included 37 males and 22 females with 1.7:1 male to female ratio. Thirty-five patients were from rural areas, whereas 24 patients were from urban areas. Thirteen patients (22%) were born to consanguineous parents.

**TABLE 1:**
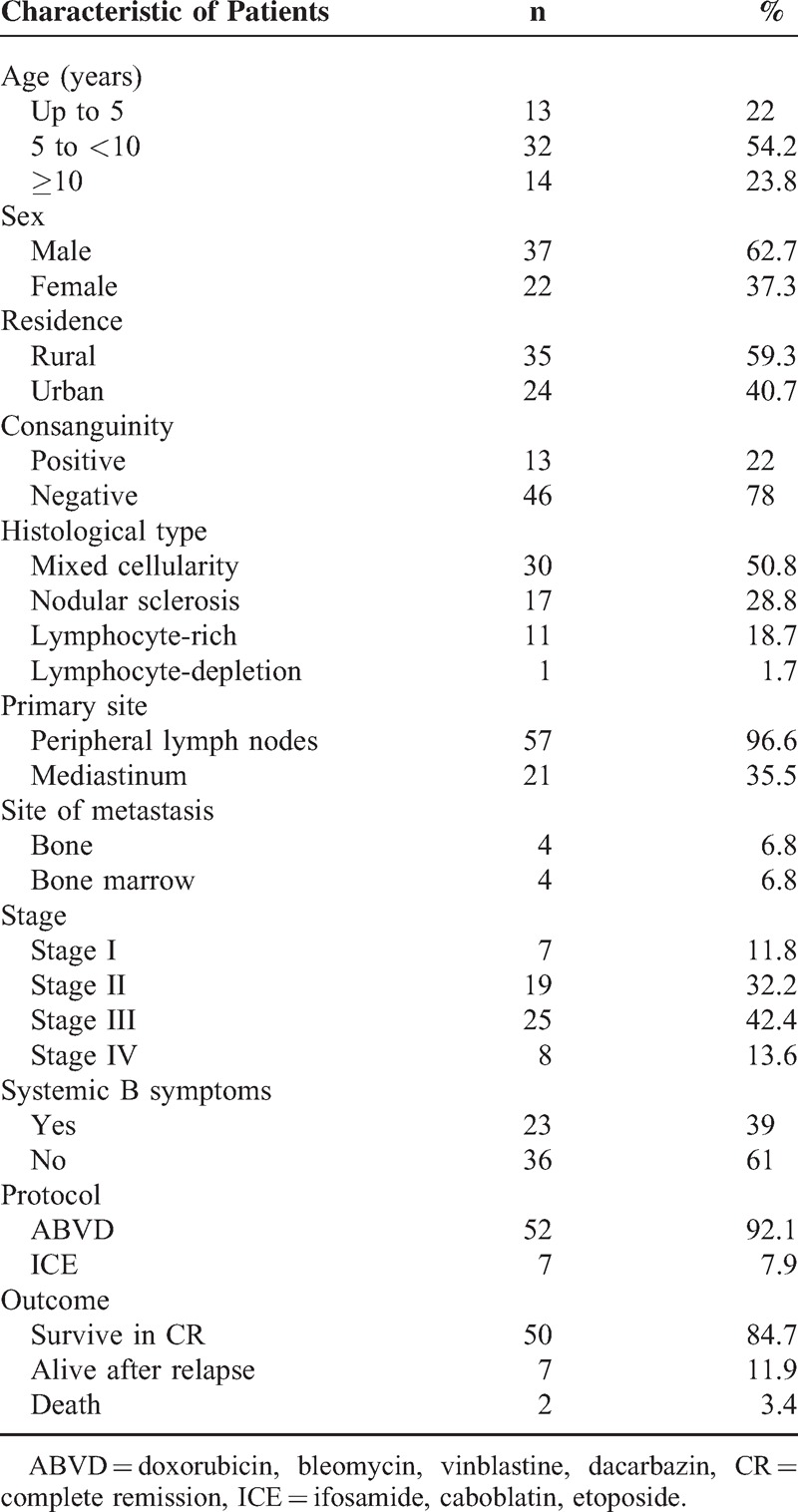
Clinical and Demographic Characteristic of the Patients

Lymphadenopathy was the most common finding among our patients, affecting 96.6% (57 patients) patients with the most common nodal sites involved were the cervical and mediastinal (n = 51, 86% and n = 39, 66% respectively). The number of involved nodal regions were <3 in 26 patients (44%), and >3 in 33 patients (56%). Two patients had extranodal disease at presentation with plumonary involvement. Twenty-three patients (39%) had B symptoms.

With relation to the histological subtypes, mixed cellularity (MC) was observed to be the predominant, affecting 50.8% (30) of the patients, followed by nodular sclerosis (NS) in 28.9% (17) of patients, lymphocyte-rich in 18.6% (11) of patients, and lymphocyte-depletion in 1.7% (1) of patients, in decreasing frequency. Bone and bone infiltration was the site of metastasis, affecting 8 patients.

All patients were staged using the Ann Arbor staging system. Thirty-three patients (55.9%) had by definition Ann Arbor stage III/IV disease and 26 (44.1%) had localized disease (stage I/II).

### Regarding Survival Outcomes

The mean follow-up period was 39.8 ± 24.1 months and range 5 to 87 months. Fifty-seven patients achieved complete remission and still alive and 2 patients (3.4%) died.

Of the patients who achieved CR, 7 (11.9%) had relapsed, treated, and still alive. The majority of relapses (5 patients) occurred within 2 years of their diagnosis. Two patients developed relapses after 5 years of their diagnosis. Most of relapses were seen in stage II (3 patients) and stage IV (3 patients), and only 1 in stage III (1 patient). The most common site of relapse was mediastinum in 4 patients. The most common histopathological type was NS (4 patients) followed by MC in 3 patients. All patients received ABVD chemotherapy as first-line therapy. Stages II and III received high-dose IFR (3500 cGY), as they had partial response following initial chemotherapy.

**FIGURE 1:**
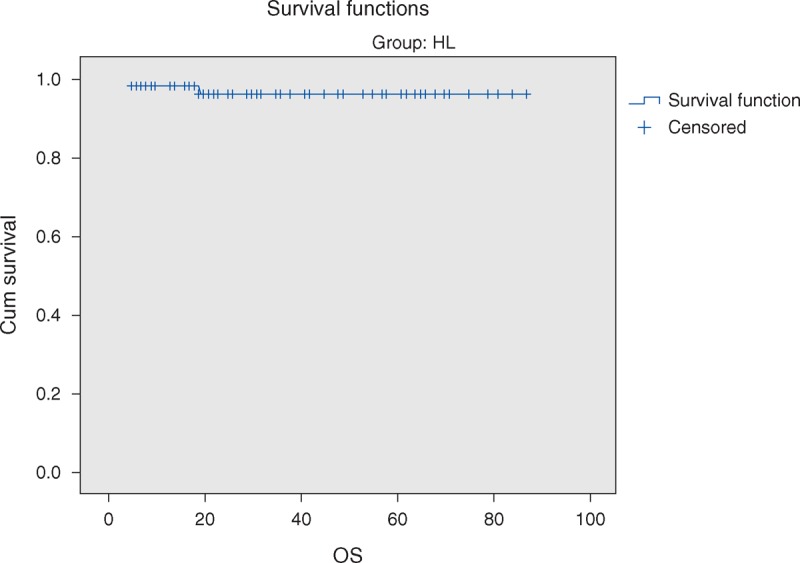
Five-year overall survival of Hodgkin lymphoma patients.

**FIGURE 2:**
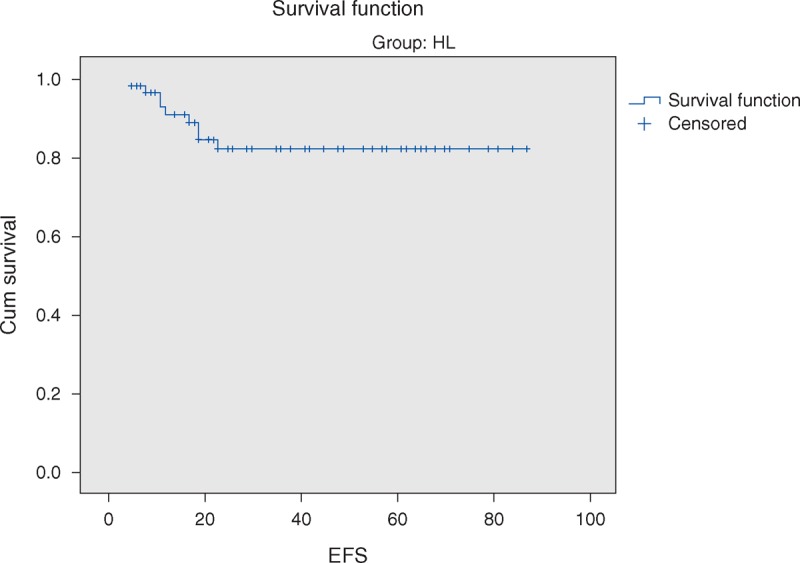
Five-year event-free survival of Hodgkin lymphoma.

**FIGURE 3:**
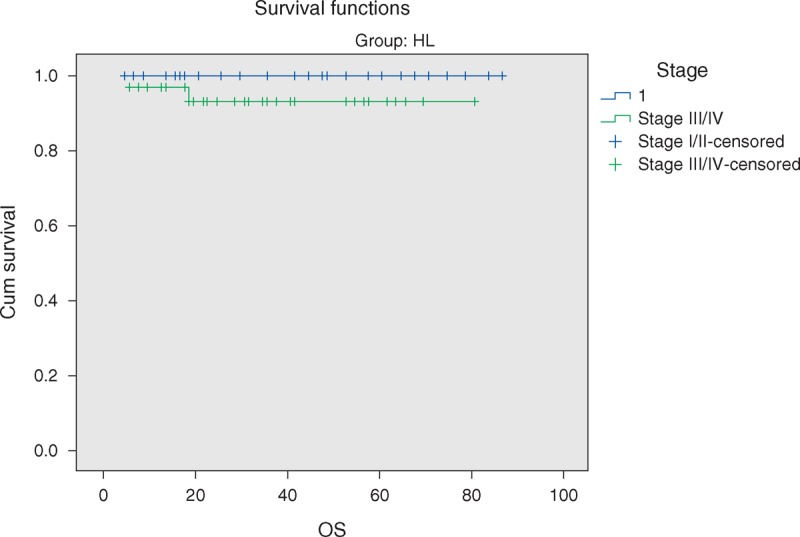
Five-year overall survival of Hodgkin lymphoma stratified by localized versus advanced clinical stage.

**FIGURE 4:**
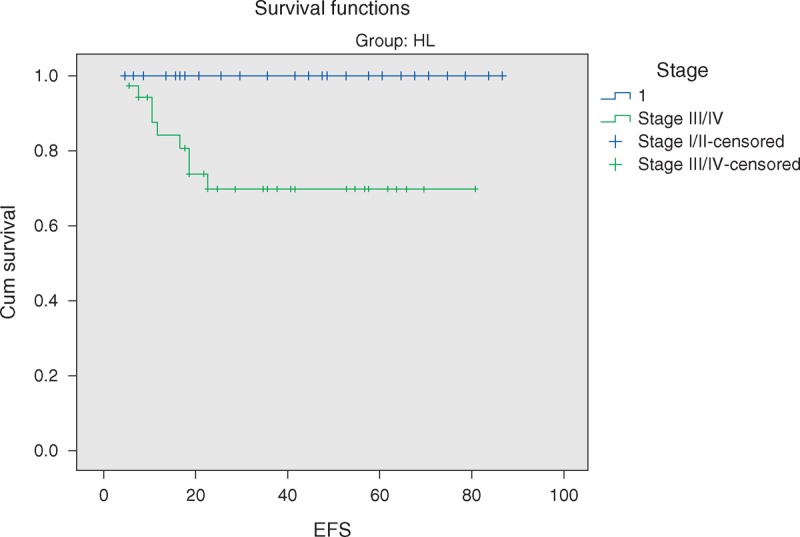
Five-year event-free survival of HL stratified by localized versus advanced clinical stage.

**FIGURE 5:**
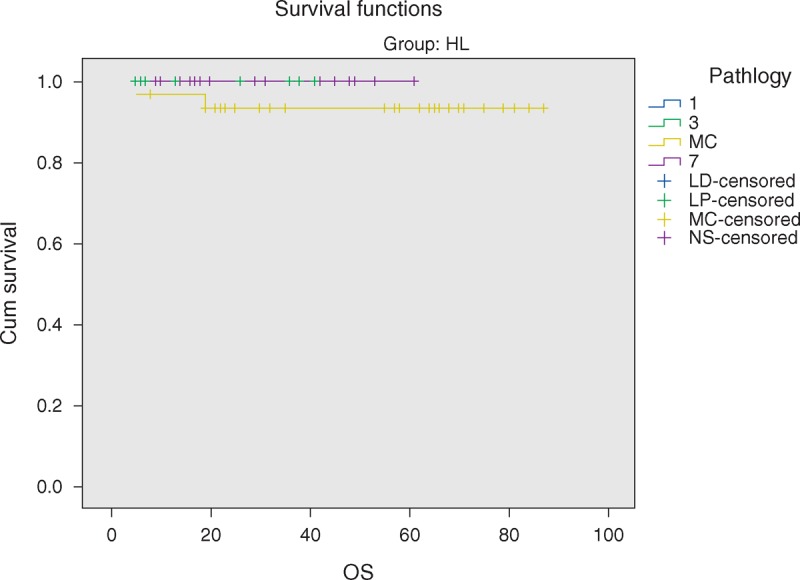
Five-year overall survival of HL according to pathological subtypes.

**FIGURE 6:**
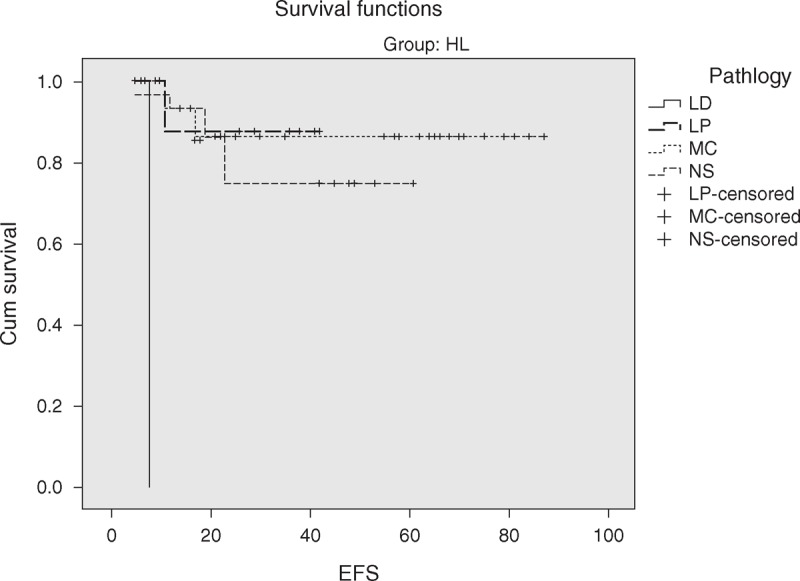
Five-year event-free survival of HL according to pathological subtypes.

The overall 5-year survival (OS) rates and event-free survival (EFS) rate of our study were 96.6% and 84.7%, respectively (Figures 1 and 2). The 5-year OS and EFS for stage I and II were 100%, whereas for stage III and IV were 93.9% and 72.7% (Figures 3 and 4). There was no statistically significant difference in the OS rate between the 4 stages (log rank-test: *P* = 0.21). Although there was a statistically significant difference in the 5-years EFS between the 4 stages and the pathological types (log rank-test: *P* = 0.006 and *P* < 0.001) (Table 2, Figures 5 and 6). Age and sex were not statistically associated with outcome (Table 2).

**TABLE 2:**
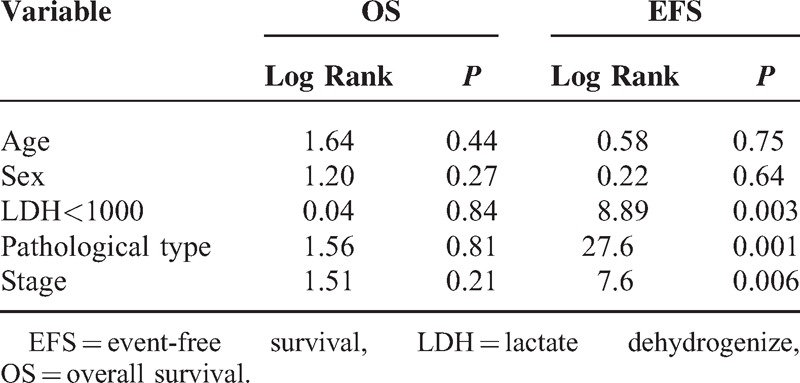
Results of Statistical Tests of Association Between OS and EFS and Study Variables in Hodgkin Lymphoma Patients

## DISCUSSION

Lymphoma in children and adolescents comprises a heterogeneous group of malignant diseases of lymphoid tissues. The various lymphoma diagnoses present with distinctive biological and epidemiological features. There are substantial variations worldwide in the incidence of childhood malignancies among which lymphoma is dominant type.^13^

The present study describes the epidemiological and clinical characteristics of 59 Egyptian children with HL who were admitted to 2 pediatric oncology units where age at presentation ranged from 3 to 14 years, with most cases (78%) were >5 years and 22% of cases were <5 years. Reports from other developing countries showed that pediatric HL occurred at a younger ages with as many as 15% to 30% of cases occurring before 5 years of age, against 5% in developed countries.^2,14–18^

In this study, male to female ratio was 1.7:1. Male predominance in HL was reported by many authors in developing and developed countries.^19–21^ Among less economically developed populations in Asia and Africa, the initial disease incidence peak occurs among boys in early childhood instead of young adulthood, indicating that risk of childhood HL may be associated with very early infection in these areas. The association of juvenile HL with early infection is substantiated by the correlation of HL risk with lower social class markers such as lower parental income and higher housing density, which promote premature exposure to common infection.^22^

The most common clinical presentation of HL patients in our study was lymphadenopathy (96.9%), which was in agreement with several authors.^18,23^

Clinical staging by the Ann Arbor staging system distinguishes 4 stages according to the extension of the disease at the moment of the diagnosis. In the present study, 11.9% of patients were having stage I disease, 32.2% stage II, 42.4% stage III, and 13.6% stage IV. In contrast, in Western countries, 75% of newly diagnosed patients have early disease at presentation (stage I–II).^14,24^ In less economically developed countries, however, more than half of the patients have advanced disease (stage III–IV),^16,18,25^ perhaps because of delayed diagnosis and referral.

The recent World Health Organization classification adopted the Revised European-American Classification of Lymphoid Neoplasms, which classifies HL into 2 disease entities according to recent clinical and biologic data: the classical HL (CHL, 95% of cases), which incorporates the 4 histology subtypes; NS, MC, lymphocyte-depleted (LD), and lymphocyte-rich (LR), and the nodular lymphocyte predominant HL (5% of cases), which is a distinct entity compared with classical disease.^26^ In children, MC, nodular lymphocyte predominant, and NS are the subtypes more commonly seen.^27^

All our patients had classical HL with MC subtype being the most common, 50.8% of the cases, followed by NS in 28.9% of cases, lymphocyte- rich HL in 18.6%, and the least common subtype was lymphocyte depletion in 1.7% of cases, which is consistent with Laskar et al^28^ in India who found that 71% of HLs were of MC and Baez et al^16^ in Nicaragua who recorded that 52.1% of HLs were of MC and 31% of NS subtype. However, several studies in Europe reported NS as the most predominant type, regardless of age.^20–21,29–30^

Distribution of HL cases over age, sex, geographic areas, and socioeconomic setting has long suggested multiple etiologically distinct entities for HL, rather than a single disease. The prevalent type in developing countries is the MC histological subtype, predominates in young children, particularly in males, mostly presents as advanced stage disease. These features could be partly explained by the pathogenesis of an etiologic role of Epstein-Barr virus (EBV) in the pathogenesis of HL. Studies have shown a causal relationship between infectious mononucleosis and subsequent EBV-positive HL.^31–33^

Childhood HL in developed countries affect mainly older children, mostly presenting as NS histologic subtype and might be explained by delayed exposure to common infectious agents, as there is increased male susceptibility to viral and bacterial infection in childhood, which is more marked in first 5 years of life.^2,34^

The vast majority of children with HL nowadays have an excellent chance of definite cure. The high curative rates as well as the prevention of late effects have to be among primary goals when managing children with HL.^35^ HL is an example of childhood cancer, which can be cured in over 90% of cases with reasonable burden of late effects.^36–37^ The 5-years OS and 5-years EFS of the current study were 96.6% and 84.7%, respectively. Pourtsidis et al^30^ in Greece reported OS of 98% and EFS of 86.2%, whereas Mutafoğlu-Uysal et al^38^ in Turkey reported 5-year OS of 96% and EFS of 72%. The 5-year survival rate for HL in European countries during the period 1985 to 1989 was on average 93% that ranges from 68% (Estonia) to 96% (Germany) and 100% (Slovenia).^39^

The 5-year EFS in our cohort was significantly lower in cases with advanced stages (III and IV) as compared with stage I and II, log rank *P* = 0.006. Similar results were reported by Dinand et al^3^ and Simth et al.^40^

We observed that patients with NS histology had significantly worse EFS versus those with other histologies. Several authors^10,40^ reported similar findings.

Beside histological subtypes, many other factors could be contributing to the outcome of HL patients. Hsu et al^41^ showed that EFS rate of patients with unfavorable disease was lower in underprivileged countries as compared with that at the industrialized countries, even when the same therapy regimen was used. Each population has its own characteristics, so any therapy regimen resulting in an excellent outcome under given conditions does not necessarily lead to the same outcome under different conditions.^6^

In general, the treatment results of children with HL have shown considerable improvement even in developing countries. The main challenge today is finding a balance between maximizing cure and minimizing the late effects.^42^ In Egypt, HL occurs at younger age, with a higher incidence of mixed cellurity and advanced disease. The outcome of HL in our 2 centers was satisfactory approaching the international rates.
